# Intercropping Maize with Faba Bean Improves Yield, Income, and Soil Fertility in Semiarid Environment

**DOI:** 10.1155/2024/2552695

**Published:** 2024-03-05

**Authors:** Tesfay Gidey, Daniel Hagos Berhe, Emiru Birhane, Yirga Gufi, Bereket Haileslassie

**Affiliations:** ^1^Department of Plant Science, College of Agriculture and Environmental Sciences, Adigrat University, P.O. Box 50, Adigrat, Ethiopia; ^2^Department of Natural Resources Management, College of Agriculture and Environmental Sciences, Adigrat University, P.O. Box 123, Adigrat, Ethiopia; ^3^Department of Land Resource Management and Environmental Protection, College of Dryland Agriculture and Natural Resources, Mekelle University, P.O. Box 231, Mekelle, Ethiopia; ^4^Institute of Climate and Society, Mekelle University, P.O. Box 231, Mekelle, Ethiopia; ^5^Faculty of Environmental Sciences and Natural Resource Management, Norwegian University of Life Sciences (NMBU), Ås, Norway; ^6^Ethiopian Forest Development, Mekelle Centre, Mekelle, Ethiopia; ^7^Tigray Agricultural Research Institute, Mekelle Soil Research Center, P.O. Box 1070, Mekelle, Ethiopia

## Abstract

Continuous adoption of improved maize varieties in the last three decades has changed farm landscapes from heterogeneity to maize homogeneity in semiarid areas of Ethiopia. This has substantially decreased maize productivity. Recently, farmers have integrated faba bean into maize-based farming systems aimed at increasing productivity. Yet, there is limited information on the effects of maize-faba bean intercropping on productivity and land-use efficiency. We studied the effects of maize intercrops with two faba bean varieties (Gora and Moti) at three different densities (25, 50, and 75%) of the recommended sole faba bean (250,000 plants ha^−1^) on yield, economic return, and some soil fertility indicators in Tigray, northern Ethiopia. Randomized complete block design with three replications was used for the experiment. The intercrops revealed that a significantly higher total grain yield, economic revenue, and land equivalent ratio (LER) over the sole cropping. Intercrops also showed higher soil organic carbon and total nitrogen compared to the preplanting soil and sole maize. Maize intercropped with the Gora faba bean variety at a density of 50% increased the total grain yields, economic return, and LER, respectively, by 13, 42, and 38% over the sole maize. The intercrop also increased soil total N by 55 and 22% compared to the preplanting soil and sole maize, respectively. Intercropping maize with faba bean significantly improved crop yield, income, land-use productivity, and some soil fertility indicators than either the sole maize or faba bean crop in the semiarid region of northern Ethiopia.

## 1. Introduction

Feeding the ever-increasing global population is currently one of the major challenges. Such population increases expanded urbanization and industrialization rapidly, thereby further shrinking cultivable lands devoted to food production [1, 2]. Looking at other economically and ecologically viable options to increase food production under the ever-increasing shrinking of lands is then crucial. Inclusion of grain legumes into cereal-based farming systems as intercrop is one of the sustainable options [2–6].

Intercropping grain legumes with cereals has been commonly practiced in the tropics to improve food security, land-use efficiency, and farm income while reducing the risks of biotic and abiotic stresses [7–10]. Intercropping provides higher yields than its sole counterpart due to the better utilization of essential growth resources such as space, water, and nutrients [6, 9, 11, 12] and reduces damage caused by pests and environmental stresses [13–16]. Moreover, legume-cereal intercropping is an important system to improve soil fertility as the component crops' ability to utilize different soil nutrient pools and the legume partner can fix atmospheric nitrogen biologically [6, 12, 17, 18].

Faba bean (*Vicia faba* L.) is one of the most commonly used grain legumes as an intercrop with maize (*Zea may* L.) [6, 11, 19]. Faba bean and maize intercropping were found to improve yield [10, 19, 20], land-use productivity [21, 22], soil fertility [6, 23, 24], and to reduce damage of weeds and pests [21, 25]. These benefits can be enhanced by the selection of appropriate management related to the component crops including planting density, time of planting, choice of compatible varieties, and planting arrangements [1, 11, 19, 26].

Maize and faba bean are the most important food crops in Ethiopia. Maize is estimated to cover an area of 2.5 million ha in the main growing season (May–October), making up 24% of the total cereal area coverage [27]. The area devoted to faba bean is also estimated to be 0.5 million ha, making up 30% of the total area coverage given for grain legumes [27]. The intercropping systems in northern Ethiopia comprise cereals with cereals, cereals with legumes, and trees with annual crops [28]. However, continuous adoption of improved maize varieties in the last three decades has changed on farms genetic diversity, increasingly leading to the growth of genetically homogeneous maize varieties. This maize monoculture practice increasingly depleted soil nutrients and created suitable conditions for damaged pests such as stem borers, thereby declining maize productivity by 15–100% [29]. Despite this, in recent years, because of the growing population pressure coupled with the increasing cost of chemical fertilizers and the strong desire to produce diverse products from the ever-shrinking land holdings, the farmers have integrated grain legumes within the maize farms. However, the benefits of faba bean integration into maize-based farming systems on crop productivity and soil fertility are still limited [26, 30]. The objectives of this study were to investigate the effects of maize intercrops with two faba bean varieties at three different population densities on: (1) grain yields and economic revenues of the component crops; (2) land-use efficiency of the cropping systems; (3) competitive relationships of the crops in intercropping; and (4) some soil fertility indicators.

## 2. Methods

### 2.1. Study Area

The field experiment was conducted in the 2019 main cropping season under rain-fed conditions at Wukro Agricultural College, Kilite-Awlielo district, Tigray, northern Ethiopia. The area is 40 km south of Mekelle city, the capital city of Tigray, and it is located at 13°43′ N latitude and 39°25′ E longitude, at an elevation of 2000 m above sea level. It is characterized by semiarid climatic conditions, with an average temperature that varied between 10 and 32°C. The total annual rainfall is between 350 and 850 mm, with the main rainy season between June and September. Soil of the area is characterized by low nitrogen content and organic matter [31]. Its texture is classed into sandy with a proportion of 9% clay, 12% silt, and 79% sand. Maize, wheat, barley, faba bean, and chickpea are the major field crops growing in the area [31, 32].

### 2.2. Experimental Design

The experiment consisted of two faba bean varieties (Gora and Moti) at three population densities (25, 50, and 75%) of the recommended sole faba bean (250,000 plants ha^−1^) were intercropped with full maize (BH-543 variety). Sole varieties of the crops were included as control treatments. The recommended 44,444 plants ha^−1^ density of maize was used in the intercrops and sole crop. The selected faba bean varieties have distinct characteristics: Gora is relatively with a short height (105–110 cm), while Moti is with a medium height (115–120 cm). Both are early maturing (taking 130–140 days from sowing to physiological maturity stage). The improved BH-543 maize variety is featured by early maturity (140–150 days), with medium height (140–180 cm), and high yield. Seeds were obtained from Tigray Agricultural Research Institute, Ethiopia.

The experimental field was well-ploughed and harrowed using a tractor for preparing a good seedbed for planting. Maize in the intercrops and sole was planted using its recommended inter- and intra-row spacing of 75 and 30 cm, respectively. For the intercrops, a plot size of 3.75 m × 5.1 m, comprising five rows of maize and within them four rows of faba bean as intercrop was used. Intrarow spacing of faba beans within the rows of maize was then adjusted according to their density: the 25% density was spaced at 39 cm, the 50% spaced at 20 cm, and the 75% was distanced only 13 cm. Sole maize used a similar plot size to the intercrops but had only five rows of maize. Three central rows of maize and two rows of faba bean with a net plot area of 7.65 m^2^ were considered for final data collection [30]. Sole faba bean was also planted using its recommended inter- and intra-row spacing of 40 and 10 cm, respectively. The plot size was 2.4 m × 5.1 m, consisting of six rows, but only two central rows with a net plot area of 2.04 m^2^ were used for data collection [33]. The experiment was laid out in a randomized complete block design with three replications. Potato was the preceding crop before the experiment.

Following the local practices, both crops were simultaneously planted in mid-June 2019. We planted two seeds per hole manually for the crops, and finally they were thinned to one vigor plant after three weeks of emergence. The intercrop and sole maize plots received the recommended rate of 41/20 kg NP ha^−1^ at the planting stage and 23 kg N ha^−1^ at the knee-height growth stage of maize (five weeks after planting) as urea and diammonium phosphate (DAP). Sole faba bean plots only received 18/20 kg NP ha^−1^ at the planting stage as DAP. Hand weeding was done at five and ten weeks after planting. All the other important agronomic managements were uniformly applied to the experimental plots, following the local recommendations. The crops were separately harvested manually in mid-October 2019.

### 2.3. Soil Sample

From each experimental plot, 300 g composite soil samples were taken twice from 0 to 30 cm depth: just before planting of the crops (preplanting soil) and then immediately after harvesting of the crops (postharvest soil). The samples were then analysed at the Shire Soil Laboratory in Ethiopia after they had been well-cleaned, air-dried, and sieved. Soil pH was measured with a pH meter in a 1 : 2.5 soil-to-water suspension. Electrical conductivity (EC) was measured by a conductivity meter in a 1 : 5 soil-to-water ratio suspension. Soil organic carbon (SOC) was determined using the Walkley and Black methods [34]. Total nitrogen (TN) and available soil phosphorus (P) were analysed using the micro-Kjeldahl and Olsen methods, respectively [35].

### 2.4. Data Collection

From each net plot area, ten maize and faba bean plants at physiological maturity were randomly selected and manually harvested to determine their fresh biomass yields. Dry biomass yields were then weighed after drying the fresh biomasses in the oven for 72 hours at temperatures of 70°C for maize and 65°C for faba bean until they achieved constant weight [36]. The whole maize and faba bean plants in the net plot were harvested, and then grains were manually threshed and adjusted to 12.5% moisture content for maize and 10% for faba bean to determine their respective grain yields [37]. The dry biomass and grain yields were converted to hectare basis (kg ha^−1^) for the statistical analysis.

### 2.5. Calculations

The relative productivity or land-use efficiency advantage of the intercrops over sole crops was calculated using the Willey [38] formula:

(1) $$\begin{array}{c} \text{LER}=\frac{{\text{intercrop yield}}_{\text{maize}}\;}{{\text{sole}}_{\text{maize }}\;}+\frac{{\text{intercrop yield}}_{\text{faba bean }}}{{\text{sole}}_{\text{faba bean }}\;}. \end{array}$$

If LER values were greater than 1.0, intercropping was considered as advantageous in productivity over the sole, whereas if LER values are less than 1.0, it was disadvantageous. Economic returns of the crops were computed using the gross monetary values (GMV) by multiplying the grain yields of the crops with their respective market prices. During November 2019, the prices for maize and faba bean were 0.35 and 0.8 USD kg^−1^, respectively, in the Wukro town market.

The competitive relationships between maize and faba bean in the intercrops were computed using the relative crowding coefficient (*k*) and aggressivity (*A*) values using the Willey [38] formulas:

(2) $$\begin{array}{c} \text{Crowding coefficient of faba bean }\left(k_{ab}\right)=\frac{{Y_{ab}\times Z}_{ba\;}\;}{\left({Y_{aa}-Y}_{ab}\right)\times Z_{ab}},\\ \text{Crowding coefficient of wheat }\left(k_{ba}\right)=\frac{{Y_{ba}\times Z}_{ab\;}\;}{\left({Y_{bb}-Y}_{ba}\right)\times Z_{ba}},\\ \text{Product of the coefficient }\left(K\right)=\left(\;k_{ab}\times k_{ba}\right),\\ \text{Aggresivity of faba bean }\left(A_{ab}\right)=\frac{Y_{ab\;}}{Y_{aa\;}\times Z_{ab}}-\;\frac{Y_{ba}}{{Y_{bb}\times Z}_{ba}},\\ \text{Aggresivity of wheat }\left(A_{ba}\right)=\frac{Y_{ba}}{Y_{bb\;}\times Z_{ba}}-\;\frac{Y_{ab}}{{Y_{aa}\times Z}_{ab}}, \end{array}$$

where *Y*_*aa* _ is the sole grain yield of maize, *Y*_*bb* _ the sole yield of faba bean, *Y*_*ab* _ is yield of maize in intercrops, *Y*_*ab*_ yield of faba bean in intercrops, *Z*_*ab*_ the sown proportion of maize, and *Y*_*ba*_ is the sown proportion of faba bean.

The other index for assessing intercrops is the system productivity index (SPI), developed by Odo [39], which standardizes the grain yield of the secondary crop, *b* (faba bean), in terms of the main crop, a (maize):

(3) $$\begin{array}{c} \text{SPI}=\frac{S_{a}}{S_{b\;}}Y_{b}+Y_{a}, \end{array}$$

where *S*_*a*_ and *S*_*b*_ are the mean grain yield of maize and faba bean in sole cropping, and *Y*_*a* _ and *Y*_*b*_ are the mean yield of maize and faba bean in the intercrops.

When the value of *K* is higher than 1.0, there is a yield advantage, if it is less than 1.0, there is a yield disadvantage. If the *A*_*ab*_ is positive, maize is dominant over faba bean in the intercrops, and if it is negative, then vice versa [38].

### 2.6. Data Analysis

A one-way analysis of variance (ANOVA) was used to analyse the collected data. The least significant difference (LSD) test at 5% probability level was used for mean comparisons whenever the ANOVA showed a significant difference. The *F*-test values for the main effects of variety and population density on yield and yield components of the crops were not significant, so only their interaction effects values were reported in the study. The Statistical Analysis Software (SAS) version 9.2 was used for the data analysis [40].

## 3. Results

### 3.1. Grain Yield and Economic Return

The cropping systems' (intercrops and sole cropping) effects on dry biomass and grain yields and GMVs of the component crops were significant (*p* < 0.05). Dry biomass and grain yields of each sole crop were greater than the respective yields in the intercrops while lower than the total yields of the intercrops (Table 1). Regardless of the faba bean varieties, increasing faba bean density from 25 to 75% in the intercrops raised faba bean dry biomass yield from 61 to 131% but had no significant effect on the dry biomass yield of maize. Increasing the density of faba bean from 25 to 75% also increased faba bean grain yield from 40 to 81% but decreased maize grain yield from 6 to 31% (Table 1). Most importantly, maize intercropped with the Gora variety at a density of 50% provided a 13 and 42% higher total grain yields and economic return, respectively, compared to the sole maize (Table 1).

### 3.2. Land-Use Efficiency and Competitive Relationships

Effects of the cropping systems on the partial and total LERs of the component crops were significant (*p* < 0.05). Increasing faba bean density from 25 to 75%, increased LERs of faba bean from 42 to 82% whereas decreased LERs of maize from 8 to 32% (Table 2). Maize intercropped with the Gora variety at density of 50% provided a 38% increase in LER compared to the other intercrop combinations (Table 2). The product of the crowding coefficients (K) of all the intercrops was greater than one, and intercropping maize with the Gora variety at a density of 50% provided the highest SPI value (Table 3) in addition to its highest total grain yield, GMV, and LER values (Tables 1 and 2). The aggressivity (*A*) values showed that the domination of faba bean over the maize crop in the intercrops (Table 3).

### 3.3. Postharvest Soil Fertility

The cropping systems significantly (*p* < 0.05) improved soil pH, SOC, and TN while decreased EC and available soil P compared to the preplanting soil. These soil parameters also had significant (*p* < 0.05) differences between the intercrops and sole cropping (Table 4). As compared to the preplanting soil and the sole maize, maize intercropped with the Gora variety at a density of 25% increased soil pH from 8.20 to 8.51 and 8.32, respectively. Maize intercropped with the Gora variety at a density of 50% provided a 61 and 23% higher SOC over the preplanting soil and sole maize, respectively (Table 4). This combination also increased the soil TN by 55 and 22%, respectively, relative to the preplant soil and sole maize. However, it decreased the available soil P by 28 and 22% when compared to the preplanting soil and sole maize, respectively (Table 4).

## 4. Discussion

Even though grain yields of the component crops in the intercrops were low compared to their respective sole crop yields, the total land productivity (total grain yields) was improved in the intercrops as supported by higher total LERs and GMVs values. In the study, the mean LER values ranging from 1.11 to 1.38 were obtained from maize intercropped with faba bean varieties at three different densities. This shows that the sole crop of each component crop requires 11 to 38% additional land to give an equal grain yield with the intercropped component crops, showing a greater land-use efficiency of the intercrops than the sole cropping. These results were similar to those of Stoltz and Nadeau [20] and Nurgi et al. [19] who found that the mean values of LER for maize-faba bean intercrops between 1.1 to 1.21 and 1.12 to 1.22, respectively. Similarly, maize-common bean intercropping provided 33 to 94% LER [41]. Besides, the study showed that a 13% higher total grain yields relative to the sole maize. This result is consistent with Nurgi et al. [19] who found 10% greater grain yield in maize-faba bean intercropping over maize monoculture due to better utilization of growth resources. The intercrops also increased the economic return of the system by 13–42% compared to the sole maize. This economic superiority of the intercrops would be linked with their higher total grain yields and LERs. Similar results were reported by Rezaei-chianeh et al. [1] who found maize intercropped with faba bean provided a 30% higher income than maize monoculture. Agegnehu et al. [42] reported faba bean and wheat intercropping offered a 0–8% higher income than the sole wheat. Nurgi et al. [19] also found a 10% higher income under maize-faba bean intercropping compared to the sole maize. Furthermore, intercropping wheat with tomatoes grown under chilling stress increased the net income by about 106% compared to the sole tomato [16].

Product of the crowding coefficients (K) of all the intercrops greater than one, showing the intercrops were superior in grain yields than the sole cropping. The positive aggressivity (*A*) values for faba bean and the negative values for maize showed that the domination of faba bean over maize in the intercrops. These results had similarity with Agegnehu et al. [7] who found that the domination of faba bean over teff (*Eragrostis tef*) grown under mixed cropping systems in central Ethiopia. Similarly, a domination of faba bean over barley was found under row intercropping systems in northern Ethiopia [43]. Wang et al. [10] also found a domination of faba bean over maize crop grown under soil moisture stress conditions. However, a domination of faba bean by wheat grown under mixed intercropping systems was found due to the latter crop similar height with faba bean's ability to compete for growth resources such as space and light [42].

Maize intercropping with faba bean significantly improved important soil fertility indicators over the preplanting soil and sole maize. For example, the intercrops had a higher SOC compared to the preplanting soil and sole maize, and this could be because of the component crops contributed much litter falls into the soil environment. These results were similar to those of Hirpa [44] and Beshir and Abdulkerim [45] who found that maize-haricot bean intercropping produced a greater amount of SOC compared to the preplanting soil and sole maize. Maize intercropped with haricot bean offered a 9% higher SOC than the preplanting soil [45]. Soils under the intercrops had higher TN than soils in the preplanting soil and sole maize. This could be because the biological N-fixation ability of faba bean would add root exudates and nodules into the soil, and the component crops produced much litter falls into the soil environment. These findings are consistent with the maize-haricot bean intercrops that raised total soil N by 18 and 4% over the preplanting soil and sole maize, respectively [44]. Intercropping maize with common beans increased soil N content by 1.6% relative to maize monoculture [5]. Barley-pea intercropping also found to add on average 22 to 30 kg N ha^−1^ into the soil pools compared to the preplanting soil [46]. The study recorded 28 and 22% lower available soil P under the intercrops relative to the preplanting soil and sole maize, respectively, and these could be due to the interspecific competition between the component crops. Such situations had also been reported in other studies, for example, maize-haricot bean intercropping reduced available soil P by 46 and 36%, respectively, compared to the preplanting soil and sole maize [36].

Maize intercropped with the Gora variety at a population density of 50% significantly produced greater total grain yields (GMV, LER, SPI, SOC, and TN) than the sole maize and the other intercrop combinations. This could be because of a better compatibility of the component crops at this specific population density on use of essential growth resources such as space, water, nutrients, and light. Similar results were reported in maize intercropped with faba bean [1] and soybean [47] at a population density of 50%.

## 5. Conclusions

The present study showed that maize intercropped with Gora and Moti faba bean varieties at 25, 50, and 75% densities of the recommended sole faba bean significantly improved total grain yields, economic revenue, and land-use efficiency when compared to the sole cropping. The postharvest soil analysis also revealed that maize intercropped with faba bean is likely to increase important soil fertility indicators. The study suggested that maize intercropped with the Gora faba bean variety at a density of 50% as a sustainable and resilient farming system to improve yield, economic return, land-use productivity, and soil fertility over the sole crops in the semiarid farming system in northern Ethiopia. However, to strengthen these findings, more research should be done at different locations in the semiarid areas in Ethiopia.

## Figures and Tables

**Table 1:** Interaction effects of intercropping maize with two faba bean (FB) varieties at three different densities on dry biomass and grain yields of the crops and gross monetary values (GMV).

Treatments	Dry biomass yield (kg·ha^−1^)		Grain yield (kg·ha^−1^)		Total grain yield (kg·ha^−1^)	GMV (USD·ha^−1^)
	Maize	FB	Maize	FB		
Sole maize	10100	—	4200a	—	4200	1470d
Sole faba bean	—	4400a	—	2000a	2000	1600c
Maize + Gora FB at 25%	9400	1300d	4050a	680c	4730	1962b
Maize + Gora FB at 50%	8000	2100c	3800ab	950bc	4750	2090a
Maize + Gora FB at 75%	8100	3000b	2800c	1230b	4030	1964b
Maize + Moti FB at 25%	9600	1200d	3370bc	610c	3980	1668c
Maize + Moti FB at 50%	8100	2100c	3240bc	1070b	4310	1990ab
Maize + Moti FB at 75%	9400	2900b	3150bc	1170b	4320	2039ab
SEM±	1040	180	230	130		39.6
LSD (5%)	NS	550	690	390		118.8
CV (%)	23.0	15.0	13.0	23.4		3.7

Means along column followed by the same letters are not significantly different (*p* < 0.05). Sole FB = averaged results of Gora and Moti varieties.

**Table 2:** Interaction effects of intercropping maize with two faba bean (FB) varieties at three different densities on partial and total land equivalent ratios (LERs) of the component crops.

Treatments	Partial and total LER values		Total
	Maize	FB	
Maize + Gora FB at 25%	0.98a	0.34b	1.32a
Maize + Gora FB at 50%	0.90abc	0.48ab	1.38a
Maize + Gora FB at 75%	0.67d	0.62b	1.29a
Maize + Moti FB at 25%	0.80cd	0.31b	1.11b
Maize + Moti FB at 50%	0.77ab	0.53a	1.30a
Maize + Moti FB at 75%	0.75bcd	0.59a	1.34a
SEM±	0.074	0.06	0.058
LSD (5%)	0.23	0.17	0.17
CV (%)	17.3	20.6	8.2

Means along column followed by the same letters are not significantly different (*p* < 0.05). Sole FB = averaged results of Gora and Moti varieties.

**Table 3:** Relative crowding coefficient (*k*), product of the coefficients (*K*), aggressivity value (*A*), and system productivity index (SPI) of maize and faba bean (FB) intercrops.

Treatments	*k* values			*A* values		
	Maize	Faba bean	*K*	Maize	Faba bean	SPI
Maize + Gora FB at 25%	6.75	2.06	13.90	−0.004	0.004	5478
Maize + Gora FB at 50%	4.75	1.81	8.60	−0.0005	0.0004	5795
Maize + Gora FB at 75%	1.50	2.13	3.20	−0.001	0.001	5383
Maize + Moti FB at 25%	1.02	1.76	1.79	−0.004	0.004	4651
Maize + Moti FB at 50%	1.69	2.30	3.89	−0.003	0.003	5487
Maize + Moti FB at 75%	2.25	1.88	4.23	−0.0003	0.0003	5607

**Table 4:** Interaction effects of intercropping maize with two faba bean (FB) varieties at three different densities on soil pH, EC (mmh·cm^−1^), SOC (g·kg^−1^), total N (g·kg^−1^), and available soil P (mg·kg^−1^).

Treatments	pH	EC	SOC	Total N	P
Preplanting soil	8.20b	0.66a	4.9b	0.43c	29.14a
Sole maize	8.32b	0.58bc	6.4ab	0.55bc	27.08ab
Sole faba bean	8.42ab	0.56c	6.5ab	0.57ab	25.94abc
Maize + Gora FB at 25%	8.60a	0.55c	6.4ab	0.55bc	22.48bc
Maize + Gora FB at 50%	8.51ab	0.52c	7.9a	0.67a	21.00c
Maize + Gora FB at 75%	8.46ab	0.55c	5.6b	0.49bc	23.64bc
Maize + Moti FB at 25%	8.52ab	0.62ab	5.5b	0.47bc	24.77abc
Maize + Moti FB at 50%	8.32ab	0.58bc	6.3ab	0.54bc	24.49abc
Maize + Moti FB at 75%	8.42ab	0.55c	6.6ab	0.57ab	22.40bc
SEM±	0.109	0.019	0.69	0.043	1.79
LSD (5%)	0.323	0.06	2.0	0.128	5.30
CV (%)	2.2	5.7	19.2	13.8	12.7

Means along column followed by the same letters are not significantly different (*p* < 0.05). Sole FB = averaged results of Gora and Moti varieties.

## Data Availability

The data used to support the findings of this study are made available from the corresponding author upon reasonable request.
